# Proteomic Profiling of Lysine Acetylation Indicates Mitochondrial Dysfunction in the Hippocampus of Gut Microbiota-Absent Mice

**DOI:** 10.3389/fnmol.2021.594332

**Published:** 2021-03-11

**Authors:** Ying Yu, Haiyang Wang, Xuechen Rao, Lanxiang Liu, Peng Zheng, Wenxia Li, Wei Zhou, Tingjia Chai, Ping Ji, Jinlin Song, Hong Wei, Peng Xie

**Affiliations:** ^1^The Ministry of Education, Key Laboratory of Laboratory Medical Diagnostics, The College of Laboratory Medicine, Chongqing Medical University, Chongqing, China; ^2^National Health Commission, Key Laboratory of Diagnosis and Treatment on Brain Functional Diseases, The First Affiliated Hospital of Chongqing Medical University, Chongqing, China; ^3^College of Stomatology and Affiliated Stomatological Hospital of Chongqing Medical University, Chongqing, China; ^4^College of Biomedical Engineering, Chongqing Medical University, Chongqing, China; ^5^Department of Neurology, Yongchuan Hospital of Chongqing Medical University, Chongqing, China; ^6^Department of Neurology, The First Affiliated Hospital of Chongqing Medical University, Chongqing, China; ^7^Department of Laboratory Animal Science, College of Basic Medical Sciences, Third Military Medical University, Chongqing, China

**Keywords:** depression, gut microbiota, lysine acetylation, germ-free, mitochondrial

## Abstract

Major depressive disorder (MDD) is a leading cause of disability around the world and contributes greatly to the global burden of disease. Mounting evidence suggests that gut microbiota dysbiosis may be involved in the pathophysiology of MDD through the microbiota–gut–brain axis. Recent research suggests that epigenetic modifications might relate to depression. However, our knowledge of the role of epigenetics in host–microbe interactions remains limited. In the present study, we used a combination of affinity enrichment and high-resolution liquid chromatography tandem mass spectrometry analysis to identify hippocampal acetylated proteins in germ-free and specific pathogen-free mice. In total, 986 lysine acetylation sites in 543 proteins were identified, of which 747 sites in 427 proteins were quantified. Motif analysis identified several conserved sequences surrounding the acetylation sites, including D^∗^Kac, DKac, KacY, KacD, and D^∗∗^Kac. Gene ontology annotations revealed that these differentially expressed acetylated proteins were involved in multiple biological functions and were mainly located in mitochondria. In addition, pathway enrichment analysis demonstrated that oxidative phosphorylation and the tricarboxylic acid cycle II (eukaryotic), both of which are exclusively localized to the mitochondria, were the primarily disturbed functions. Taken together, this study indicates that lysine acetylation alterations may play a pivotal role in mitochondrial dysfunction and may be a mechanism by which gut microbiota regulate brain function and behavioral phenotypes.

## Graphical Abstract

Lysine acetylation changes may play a pivotal role in the mitochondrial.

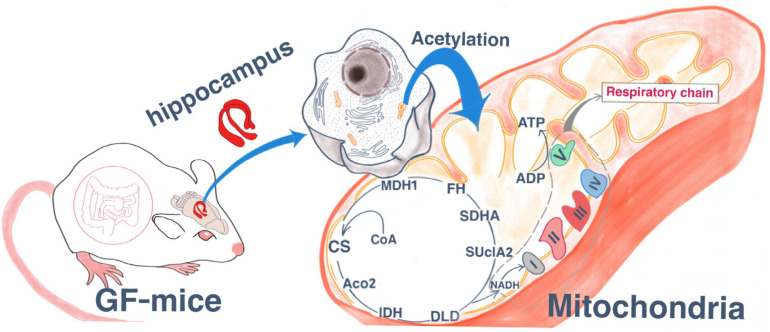

## Introduction

Major depressive disorder (MDD) is a common mental disorder that is a leading cause of permanent disability worldwide, with over 264 million people of all ages affected globally (Gbd 2017 Disease and Injury Incidence and Prevalence Collaborators, 2018). MDD is caused by gene-environment interactions (Sullivan et al., 2000). There is growing evidence to suggest that gut microbiota are an important environmental factor that can shape the physiology of the brain through the gut–microbiota–brain axis. Thus, gut microbiota alterations may be a key trigger for the development of many neuropsychiatric diseases (Cryan and Dinan, 2015).

The microbiome plays an important role in neural development and can lead to changes in gene expression in key regions of the brain, disrupting normal social and cognitive behavior in mice (Arentsen et al., 2015; Desbonnet et al., 2015; Zheng et al., 2016). Our previous studies indicate a causal role of gut microbiota dysbiosis in the onset of depression (Zheng et al., 2016; Huang et al., 2019). Furthermore, gut microbiota dysbiosis can lead to alterations in the glucocorticoid receptor pathway, cyclic AMP-responsive element-binding protein (CREB) signaling, and hypothalamic-pituitary-adrenal axis in the hippocampus (Zeng et al., 2016; Luo et al., 2018), hypothalamus (Huo et al., 2017), and olfactory bulb (Huang et al., 2019) of mice. However, the molecular mechanisms of gut microbiota-brain interactions remain unclear.

Currently, the interaction between genes and environmental factors is an important focus of depression research. Epigenetics refers to heritable changes that do not involve changes in the DNA sequence (Holliday, 1987; Ogino et al., 2013). Epigenetic changes regulate gene expression and cell differentiation and development in almost all tissues, including the brain (Horgusluoglu et al., 2017). For example, recent studies have suggested that epigenetic changes affecting hippocampal neurogenesis are implicated in learning and memory (Hsieh and Eisch, 2010; Horgusluoglu et al., 2017). In addition, many studies have reported that the microbiome and the epigenome interact, and gut microbiota activity can modify the host’s epigenome, thereby affecting gene expression (Gao et al., 2009; Alenghat et al., 2013; Majnik and Lane, 2015). Epigenetic mechanisms are involved in several functions, such as neurogenesis, neuronal plasticity, learning, and memory, as well as in diseases such as depression, addiction, schizophrenia, and cognitive dysfunction (Stilling et al., 2014; Landgrave-Gómez et al., 2015). Notably, there is increasing research emphasis on the role of epigenetic mechanisms in shaping the brain and behavior. To date, however, the role of epigenetics in host–microbe interactions is poorly understood. Lysine acetylation is a reversible and highly regulated post-translational modification (PTM) that is widespread in almost all organisms. It has important roles in the regulation of protein function, involving diverse metabolic pathways to not only regulate nuclear function but also to control cytoplasmic and mitochondrial functions (Choudhary et al., 2009; Zhao et al., 2010). Many proteomic studies have reported that acetylation occurs at thousands of sites throughout eukaryotic cells, and that the human proteome contains at least 2,500 acetylated proteins (Kim et al., 2006; Choudhary et al., 2009; Vancura et al., 2018). In human cells, acetylated proteins play important roles in various cellular processes, such as chromatin remodeling, the cell cycle, RNA metabolism, cytoskeletal dynamics, membrane transport, and key metabolic pathways, such as glycolysis and gluconeogenesis, and the citric acid cycle (Choudhary et al., 2009; Zhao et al., 2010). Generally speaking, protein acetylation can not only activate and inhibit the enzyme activities of proteins but can also inhibit the interactions between proteins (Guarente, 2011; Galdieri et al., 2014; Drazic et al., 2016; Salminen et al., 2016). Acetylation of histones can affect chromatin structure and gene expression (Eberharter and Becker, 2002). Lysine acetylation is regulated by two groups of enzymes with opposing activities: lysine acetyltransferases and lysine deacetylases (Yang and Seto, 2007). Here, we aimed to investigate whether there are epigenetic changes in acetylation under conditions of absence of gut microbiota.

Germ-free (GF) mice, which are not exposed to microbes during birth or growth, can be used as a model for understanding the effects of gut microbiota on their hosts’ physiological behavior (Grover and Kashyap, 2014). In recent studies, the gut microbiota have been reported to influence the function and behavior of the brain through the “microbiota–gut–brain axis” (Bercik et al., 2011; Diaz Heijtz et al., 2011; Neufeld et al., 2011; Cryan and Dinan, 2012; Clarke et al., 2013). GF mice are a powerful research tool and have been widely used to evaluate the effects of gut microbes (Mayer et al., 2015; Luczynski et al., 2016). Furthermore, compared with specific pathogen-free (SPF) mice, GF mice are reported to have less non-spatial memory, social motivation, and anxiety (Gareau et al., 2011; Neufeld et al., 2011; Cryan and Dinan, 2012; Desbonnet et al., 2014). In our previous studies, we reported that GF mice exhibit antianxiety- and antidepression-like behaviors compared with their conventionally raised SPF mouse counterparts (Zheng et al., 2016; Luo et al., 2018). Studies of GF mice have indicated that specific microbiota can affect the physiology and neurochemistry of the central nervous system (Smith, 2015). In addition, GF mice with an absence of gut microbiota exhibit neurological deficits in learning, memory, and recognition (Gareau et al., 2011; Foster et al., 2017).

In the present study, we used tandem mass tag (TMT) labeling and Kac affinity enrichment followed by high-resolution liquid chromatology (LC)-mass spectrometry (MS)/MS analysis. This quantitative lysine acetylome analysis procedure was used to identify global changes in lysine acetylation in hippocampal tissue from germ-free (GF) and specific pathogen-free (SPF) mice. Bioinformatic analyses were then performed to reveal the underlying mechanisms by which gut microbiota regulate brain functions and behavioral phenotypes. Our findings indicate thatacetylated proteins are involved in TCA cycle and oxidative phosphorylation and are mainly located in mitochondria (Graphical Abstract).

## Materials and Methods

### Animals

Male GF Kunming mice and SPF Kunming mice (*n* = 8 per group, 8 weeks old) were provided by the Experimental Animal Center of the Third Military Medical University (Chongqing, China) and bred at the Experimental Animal Center of the Third Military Medical University (GB 14922.2-2011). GF Kunming mice were rederived by sterile cesarean section. The germ-free status of mice was ensured by testing the feces and skin according to Chinese Laboratory animal—Microbiological standards and monitoring (GB-T 14926A-2001), as well as PCR analysis using a universal primer for the V3 region of the bacterial 16S rRNA gene (Yuan et al., 2011). The GF mice were kept in flexible film gnotobiotic isolators. The SPF mice were housed in a barrier facility, certified by the International Association of Laboratory Animal Care Evaluation and Certification (Laboratory Animal Facility of Tsinghua University). Mice were housed in standard autoclaved polypropylene cages in groups of five animals under a 12 h light/dark cycle (lights on from 07:30 to 19:30), a constant temperature of 21–22°C, and a relative humidity of 55% ± 5%. Both water and autoclaved standard mice chow of the same formulation were available *ad libitum* to all animals. This study was approved by the Ethics Committee of Chongqing Medical University and conducted in accordance with the National Institutes of Health Guide for the Care and Use of Laboratory Animals (NIH Publication No. 80-23).

### Sample Collection and Preparation

Mice were anesthetized with 10% chloral hydrate and immediately decapitated. The hippocampus was then dissected out on an ice-cold plate and frozen in liquid nitrogen. Brain tissue was stored at −80°C until the assays were performed.

Each hippocampal sample [GF group (*n* = 8) and SPF group (*n* = 8)] was first ground with liquid nitrogen, and then transferred to a 5 mL centrifuge tube and sonicated three times on ice using a high-intensity ultrasonic processor (Scientz) in lysis buffer [8 M urea, 2 mM EDTA, 3 μM trichostatin A (TSA), 50 mM nicotinamide (NAM), 10 mM dithiothreitol (DTT), and 1% Protease Inhibitor Cocktail III]. The mixture was then centrifuged (20,000 × *g*, 4°C for 10 min) to obtain the supernatant. Subsequently, the protein in the supernatant was precipitated with cold 15% tricarboxylic acid (TCA) for 2 h at −20°C. After centrifuging at 4°C for 10 min, the supernatant was discarded. The remaining precipitate was washed three times with cold acetone. The protein was then redissolved in buffer (8 M urea, 100 mM TEAB; pH 8.0) and the protein concentration was determined using a 2-D Quant Kit (GE Healthcare) according to the manufacturer’s instructions.

### Trypsin Digestion and High-Performance LC (HPLC) Fractionation

For digestion, the protein solution was reduced with 10 mM DTT for 1 h at 37°C and alkylated with 20 mM iodoacetamide for 45 min at room temperature in the dark. For trypsin digestion, the protein sample was diluted by adding 100 mM TEAB to urea at a concentration <2 M. Finally, trypsin (Promega) was added at a 1:50 trypsin-to-protein mass ratio for the first digestion overnight, and a 1:100 trypsin-to-protein mass ratio for a second 4 h digestion.

The sample was then fractionated using high-pH reverse-phase HPLC on an Agilent 300 Extend C18 column (5 μm particles, 4.6 mm ID, 250 mm length). Briefly, the peptides were first separated with a gradient of 2–60% acetonitrile in 10 mM ammonium bicarbonate, pH 10, over 80 min into 80 fractions. The peptides were then combined into eight fractions and dried by vacuum centrifugation.

### Affinity Enrichment of Acetylated Peptides

To enrich Kac peptides, tryptic peptides dissolved in NETN buffer (100 mM NaCl, 1 mM EDTA, 50 mM Tris-HCl, 0.5% NP-40; pH 8.0) were incubated with pre-washed antibody beads (PTM Biolabs) at 4°C overnight with gentle shaking. The beads were washed four times with NETN buffer and twice with double-distilled H_2_O. The bound peptides were eluted from the beads with 0.1% TFA. The eluted fractions were then combined and vacuum dried. The resulting peptides were cleaned with C18 Zip Tips (Millipore) according to the manufacturer’s instructions, followed by LC-MS/MS analysis.

### LC-MS/MS

The enriched peptides were dissolved in 0.1% FA and directly loaded onto a reversed-phase pre-column (Acclaim Pep Map 100, Thermo Scientific). Peptide separation was performed using a reversed-phase analytical column (Acclaim Pep Map RSLC, Thermo Fisher Scientific). The gradient was comprized of an increase from 6 to 22% of solvent B (0.1% FA in 98% ACN) for 24 min, from 22 to 35% for 8 min, climbing to 80% in 5 min, and then holding at 80% for the last 3 min, all at a constant flow rate of 300 nL/min on an EASY-nLC 1000 UPLC system. The resulting peptides were analyzed using the Q Exactive^TM^ Plus Hybrid Quadrupole-Orbitrap Mass Spectrometer (Thermo Fisher Scientific) in triplicate.

The peptides were subjected to the NSI source followed by MS/MS in the Q Exactive^TM^ Plus coupled online to the UPLC. Intact peptides were detected in the Orbitrap at a resolution of 70,000. Peptides were selected for MS/MS using an NCE setting of 30. Ion fragments were detected in the Orbitrap at a resolution of 17,500. A data-dependent procedure that alternated between one MS scan followed by 20 MS/MS scans was applied for the top 20 precursor ions (above a threshold ion count of 5e3 in the MS survey scan), with a 15.0 s dynamic exclusion. The applied electrospray voltage was 2.0 kV. Automatic gain control was used to prevent overfilling of the orbitrap, and 5e4 ions were accumulated for the generation of MS/MS spectra. For the MS scans, the m/z scan range was 350 to 1,800. The fixed first mass was set as 100 m/z.

### Database Search

The resulting MS/MS data were processed using MaxQuant with the integrated Andromeda search engine (Cox et al., 2011) (v.1.4.1.2). Tandem mass spectra were searched against Uniprot/Swissprot_Mouse FASTA (16,717 sequences; downloaded 8/2015), concatenated with a reverse decoy database. Trypsin/P was specified as the cleavage enzyme, allowing up to four missing cleavages, five modifications per peptide, and five charges. Mass error was set to 10 ppm for precursor ions and 0.02 Da for fragment ions. Carbamidomethylation of cysteine was specified as the fixed modification, and oxidation of methionine, acetylation of lysine, and acetylation of protein N-terminals were specified as the variable modifications. The false discovery rate thresholds for proteins, peptides, and modification sites were set below 1%. The minimum peptide length was set at 7. For the quantification method, TMT-6-plex was selected. All other parameters in MaxQuant were set to their default values. The site localization probability was set as >0.75. The quantitative *p*-value was calculated using the two-sample, two-sided *t*-test, and the enrichment statistical method was Fisher’s exact test.

### Bioinformatic Analysis

Differentially expressed acetylation proteins were identified on the basis of the following criteria: *p* < 0.05 and fold change >1.2 or <0.83. These parameter thresholds were set to be consistent with previous studies (Ren et al., 2013; Wu et al., 2016; Huang et al., 2019). Soft motif-x was used to analyze the conserved sequences of amino acids in specific positions of modify-21-mers (10 amino acids upstream and downstream of the site) in all protein sequences (Chou and Schwartz, 2011). All of the database protein sequences were used as background database parameters, and the other parameters were set to their defaults. Secondary structures were predicted using NetSurfP (Klausen et al., 2019) and the statistical analysis was performed using the *t*-test. We used WoLF PSORT, a subcellular localization predication software, to predict subcellular localization (Horton et al., 2007), and the parameter is default and the predictive background selected animal. For the differently expressed proteins, gene ontology (GO) and Kyoto Encyclopedia of Genes and Genomes (KEGG) pathway analyses were performed using OmicsBean (Iqbal et al., 2018)^1^. Hypergeometric algorithms were used for the enrichment analysis of KEGG pathway and GO enrichment analyses, and terms with *p* < 0.05 were taken to be statistically significant. The differentially expressed proteins were then uploaded into Ingenuity Pathway Analysis (IPA) software (Ingenuity Systems)^2^ @@ to explore the disrupted functions (Krämer et al., 2014). The select scoring method was the Fisher’s exact test *p*-value [*p* < 0.05, *p* score = −log10 (*p*-value)]. Protein–protein interaction networks of the lysine-acetylated and domain functional descriptions were analyzed using the Search Tool for the Retrieval of Interacting Genes/Proteins (STRING) database (v9.1). They were then visualized using Cytoscape software (v3.1.0) with the MCODE App toolkit (Bader and Hogue, 2003; Shannon et al., 2003; Szklarczyk et al., 2015).

## Results

### Proteome-Wide Analysis of Lysine Acetylation Sites and Proteins in Mice With an Absence of Gut Microbiota

**FIGURE 1:**
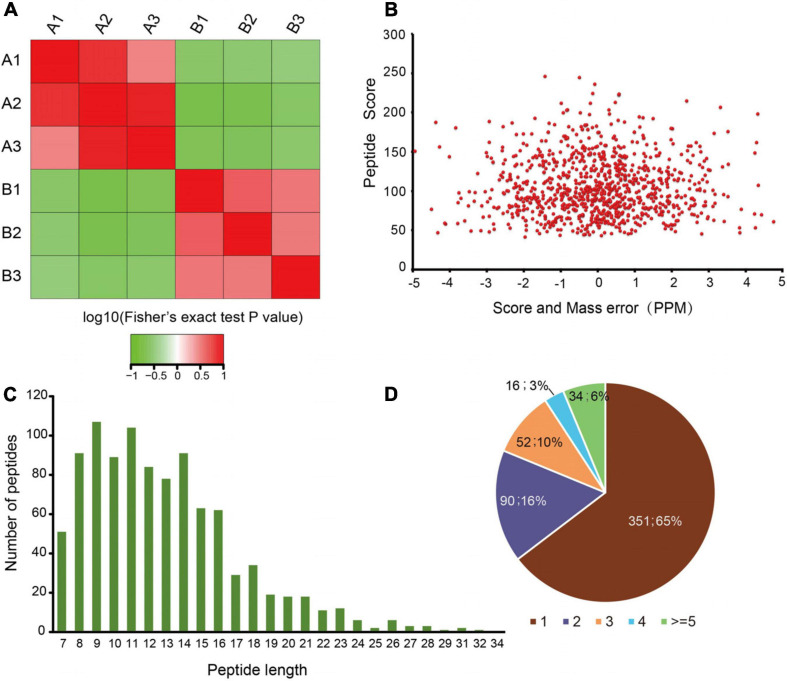
Proteome-wide identification of lysine acetylation sites in mice with an absence of gut microbiota. **(A)** Reproducibility analysis of three repeated trials using Pearson’s correlation coefficient. **(B)** Mass error distribution of all of the identified peptides. **(C)** Peptide length distribution. **(D)** Pie chart illustrating the number of lysine acetylation sites per protein.

To quantify dynamic changes of the whole acetylome in mice with an absence of gut microbiota, an integrated approach was used, involving TMT labeling, HPLC fractionation, Kac antibody affinity enrichment, and LC-MS/MS. The repeatability analysis of the three repeated experiments indicated that the experimental data were reproducible (Figure 1A). In total, 986 lysine acetylation sites in 543 protein groups were identified, of which 747 sites in 427 proteins were quantified (Supplementary Table S1). This finding implies that protein acetylation may play an important role in various physiological processes. The MS data validation is shown in Figure 1. We first checked the mass error of all of the identified peptides. The distribution of the mass error was near 0 and most were less than 5 ppm, which indicates that the mass accuracy of the MS data fit the requirement (Figure 1B). Second, the length of most peptides was distributed between 8 and 20, which is in accordance with the properties of tryptic peptides (Figure 1C) and indicates that sample preparation reached the standard. These proteins contained different numbers of acetylation sites (Figure 1D). Of the acetylated proteins, 65% contained only one acetylation site, while the percentages with two, three, and four modification sites were 16, 10, and 3%, respectively. Just 6% of the acetylated proteins carried five or more identified acetylation sites.

### Analysis of Acetylated Lysine Motifs in Mice With an Absence of Gut Microbiota

**FIGURE 2:**
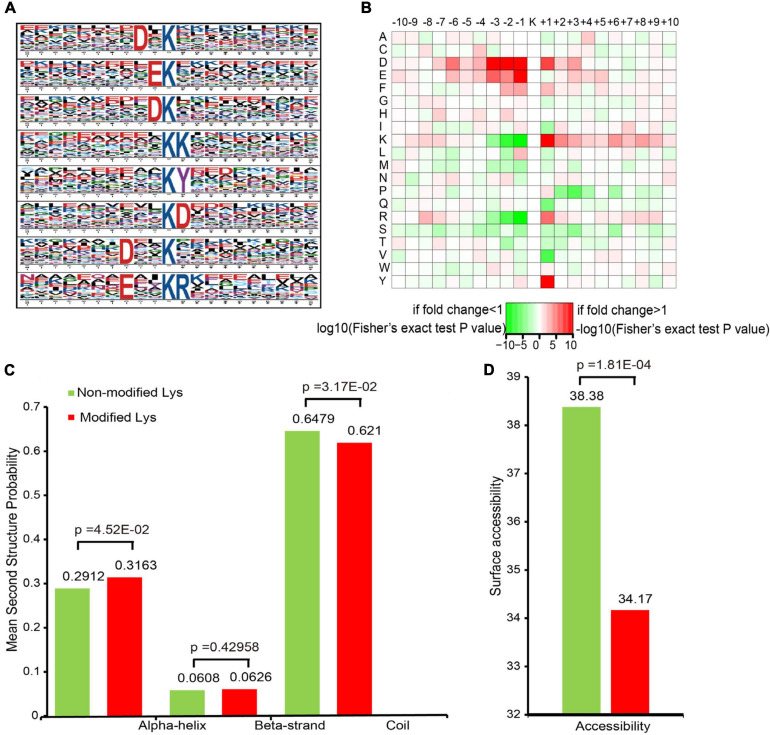
Motif analysis of all of the identified sites. **(A)** Acetylation motifs and the conservation of acetylation sites. **(B)** Heat map of the amino acid compositions of the acetylation sites. **(C)** Probabilities of lysine acetylation in different protein secondary structures. **(D)** Predicted surface accessibility of acetylated sites.

To evaluate the properties of the sequences surrounding acetylation sites in mice, we compared the 10 flanking amino acid frequencies upstream and downstream of the acetylation site. We defined eight significantly enriched acetylation site motifs from the quantifiable lysine-acetylated sites: D^∗^Kac, EKac, DKac, KKac, KacY, KacD, D^∗∗^Kac, and E^∗∗^KacR (Figure 2A and Supplementary Table S2), where Kac indicates the acetylated lysine and ^∗^ indicates a random amino acid residue. An enrichment of tyrosine (Y) and aspartic acid (D) residues was found downstream of the acetylated lysine, whereas aspartic acid (D) and glutamic acid (E) were found upstream of the modification site. Notably, acidic amino acids, such as D on the −3/−2/−1 positions and E on the −1 position, were observed among these motifs. As previously reported, lysine acetylation had a tendency toward having negatively charged acidic amino acid residues in the immediate surroundings of the modified site (Lundby et al., 2012). Additionally, a heat map of the amino acid composition around the acetylation sites show that the highest frequencies in the motifs are D in positions −3 to −1, and E (Figure 2B). These data suggest that acidic amino acids around lysine may be functionally important for acetylation.

To elucidate the relationship between lysine acetylation and the local secondary structures of acetylated proteins, we performed secondary structure predictions. Most acetylation sites were located on the coil (62.1%) in mice, followed by the α-helix (31.63%) and the β-strand (6.26%) (Figure 2C). The surface accessibilities of acetylated lysine sites were also evaluated. Approximately 34.17% of the acetylated lysine sites were exposed to the protein table (Figure 2D). These findings suggest that lysine acetylation may occur preferentially in coil structures in mice.

### Characterization of the Lysine Acetylome in Mice With an Absence of Gut Microbiota

**FIGURE 3:**
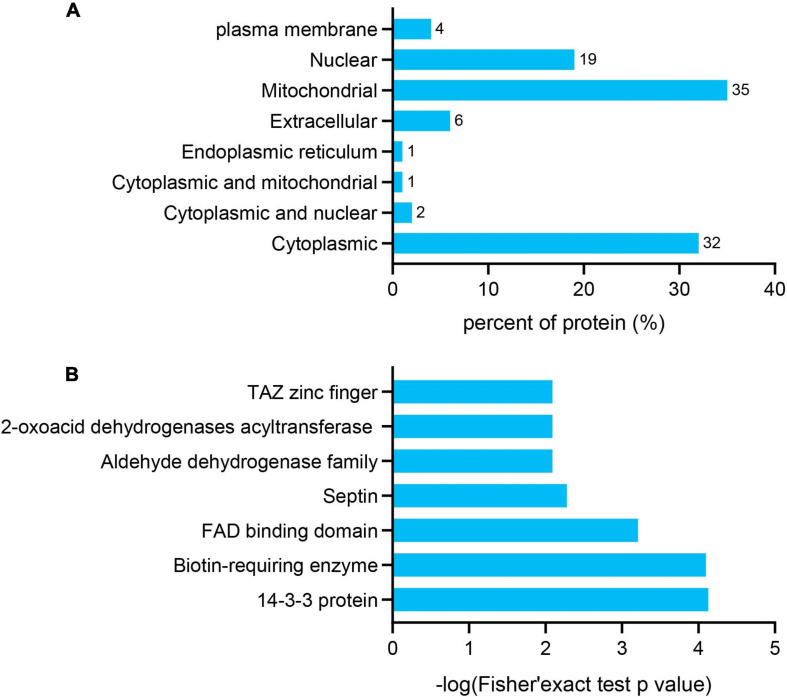
Functional enrichment of differentially quantified proteins. **(A)** Subcellular localizations of the acetylated proteins. **(B)** Protein domain enrichment analysis of the identified proteins.

The subcellular localization, domain, and GO and KEGG pathways were used to analyze the biological functions and relevant networks of differentially expressed lysine acetylation proteins, which were merged and filtered using the criteria of *p* < 0.05 and a 1.2-fold change. We also analyzed the subcellular localizations of the differentially expressed acetylated proteins. They were mainly distributed in the mitochondria (35%), cytoplasm (32%), and nucleus (19%) (Figure 3A).

**FIGURE 4:**
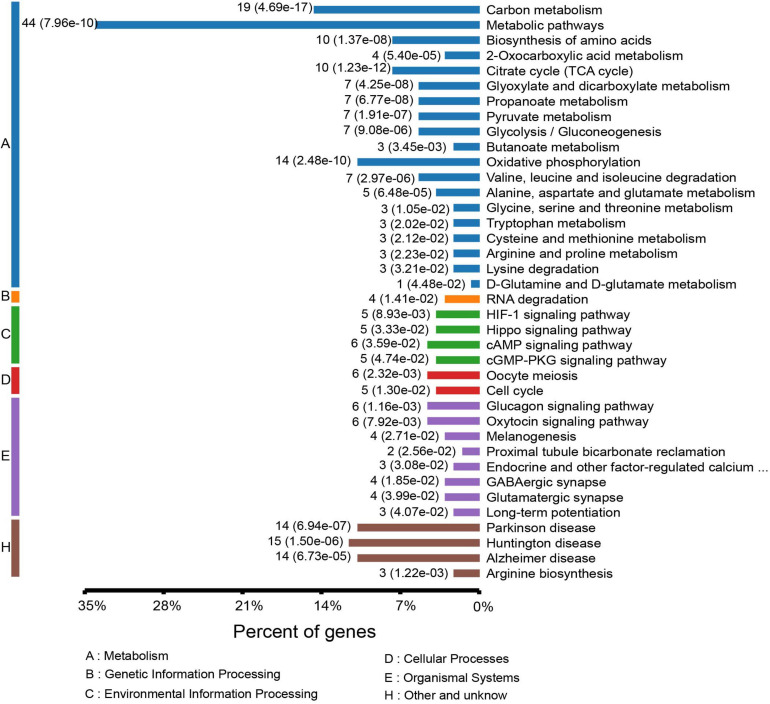
KEGG pathway-based enrichment analysis of the differentially expressed acetylated proteins.

To gain a better understanding of the lysine acetylome in the mouse, we analyzed the GO functional classifications of differentially expressed proteins on the basis of their biological process, molecular function, and cellular component categories (Supplementary Figure S1 and Supplementary Table S3). In the biological process, cellular component, and molecular function categories, the acetylated proteins were metabolized in small molecules (*p* = 1.97e–19) and mitochondria (*p* = 2.23e–37), and the structural constituents of oxidoreductase activity (*p* = 7.98e–10) were significantly enriched. These findings suggest that acetylated proteins may play essential roles in various cellular processes. To further understand the functions of the differentially expressed acetylated proteins, we conducted functional enrichment of KEGG pathway analysis. These proteins were enriched in carbon metabolism (*p* = 4.69e–17), the TCA cycle (*p* = 1.23e–12), and oxidative phosphorylation (*p* = 2.48e–10) (Figure 4 and Supplementary Table S4). Taken together, these results indicate that mitochondria-related proteins are most likely to be acetylated in mice and that absence of gut microbiota may cause mitochondrial dysfunction through protein acetylation. Domain enrichment analysis revealed that translation protein 14-3-3 domains and biotin-requiring enzyme domains have a higher tendency to be acetylated (Figure 3B and Supplementary Table S5).

### IPA Analysis of Acetylated Proteins

**FIGURE 5:**
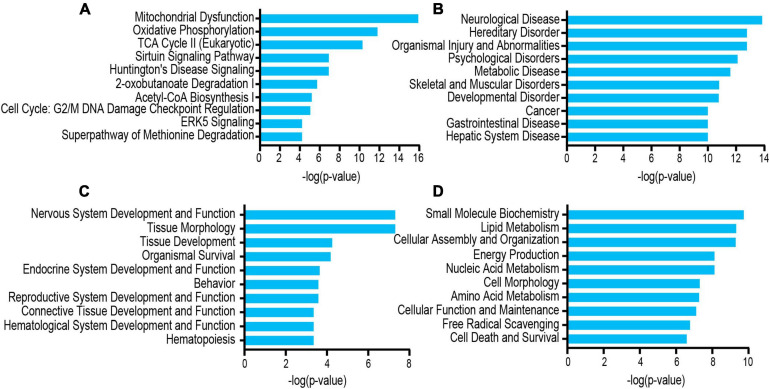
Functional and pathway analyses of the differentially expressed proteins using IPA. **(A)** Top related canonical pathways in IPA. **(B)** Disease and disorders. **(C)** Physiological system development and functions. **(D)** Molecular and cell functions.

The differentially acetylated proteins were further analyzed using the IPA database, which revealed that these proteins were significantly enriched in neurological disorders. To better understand the differentially expressed proteins, the following three major types were used for this analysis: “disease and disorder” (Figure 5B), “physiological system development and functions” (Figure 5C), and “molecular and cell functions” (Figure 5D). For the “diseases and disorders,” neurological disease was significantly observed. For the “molecular and cell functions” and “physiological system development and functions,” there were also many concepts related to depression research, such as nervous system development and function, tissue morphology, lipid metabolism, cellular assembly and organization, and energy production. Additionally, the top 10 statistically significant pathways among the GF and SPF groups included those of mitochondrial dysfunction, oxidative phosphorylation, TCA cycle II (eukaryotic) and the Sirtuin signaling pathway (Figure 5A). These pathways were consistent with those of the KEGG pathway analysis. Of the significant canonical pathways, many pathways were strongly associated with energy metabolism and production, warranting further study.

### Analysis of the Protein–Protein Interaction Networks of Acetylated Proteins in Mice With an Absence of Gut Microbiota

**FIGURE 6:**
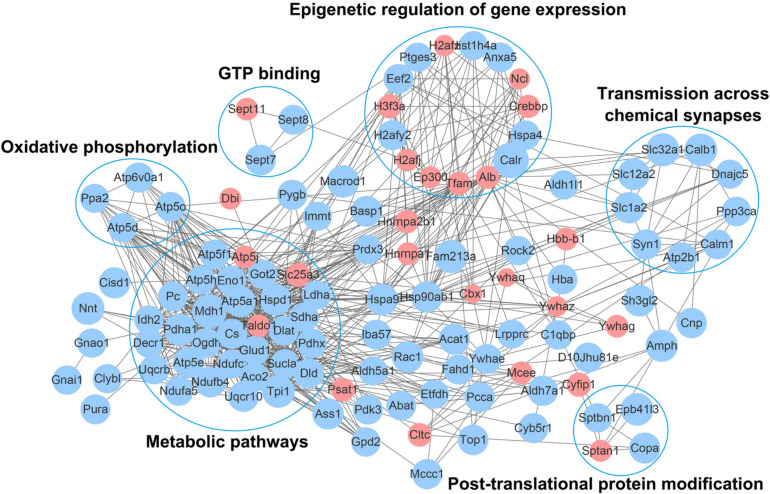
Network diagram showing interactions between the differentially expressed acetylated proteins, extracted from the STRING database. The nodes represent proteins, and the colors of the nodes represent the upregulation or downregulation of these proteins; blue represents upregulated proteins, while red represents downregulated proteins. The size of each node represents the fold-change value of the protein.

To further understand the function of acetylation in mice with an absence of gut microbiota, a protein–protein interaction network was established. A total of 178 differentially expressed acetylated proteins were uploaded into the protein network database, and the interaction networks were determined with a minimum required interaction score of 0.400. The global network graph of these interactions is shown in Figure 6 and Supplementary Table S6. The nodes represent proteins, and the colors of the nodes represent the upregulation or downregulation of these proteins; blue represents upregulated proteins, while red represents downregulated proteins. The size of each node represents the fold-change value of the protein. In this study, a protein–protein interaction network containing 117 nodes and 665 edges was constructed. To characterize the protein complexes among the acetylated proteins, the protein–protein interaction network was analyzed for highly connected nodes using MCODE. Six highly connected clusters were identified: Metabolic pathways, Epigenetic regulation of gene expression, Oxidative phosphorylation, Transmission across chemical synapses, Post-translational protein modification, and GTP binding.

## Discussion

Although lysine acetylation is an extensive and highly conserved post-translational modification involved in multiple biological functions (Choudhary et al., 2009), the role of this modification in mice with gut microbial absence is presently unclear. In the current study, we focused on global acetylation changes in hippocampal tissue from GF and SPF mice using a combination of affinity enrichment and high-resolution LC-MS/MS analyses. A total of 543 acetylated proteins with 986 lysine acetylation sites were identified in mice with an absence of gut microbiota. To better understand the functions of these acetylated proteins, we performed extensive bioinformatic analyses. Our findings indicate that these acetylated proteins in mice with an absence of gut microbiota are associated with diverse biological processes and are especially associated with mitochondria.

Recent studies have reported that non-spatial memory, social motivation, and anxiety disorders are reduced in GF mice compared with conventionally bred SPF-like mice (Neufeld et al., 2011; Clarke et al., 2013; Desbonnet et al., 2014; Zheng et al., 2016). We have previously reported that an absence of gut microbiota induces decreased immobility time in the forced swim test and an increased percentage of center distance in the open-field test, which suggests a potential link between gut microbiota and depression- and anxiety-like behaviors (Zheng et al., 2016; Luo et al., 2018).

In the present study, our data provide evidence that site-specific lysine acetylated proteins are involved in multiple physiological functions. We revealed that some proteins related to energy production were modified with acetyl groups, which implies an important regulatory role of lysine acetylation in this process. The acetylation of relevant proteins in mitochondria was abundant, and the average number of modification sites in energy-producing mitochondria was high. Mitochondrial acetylation is mainly controlled by nicotinamide adenine dinucleotide (NAD) and -dependent deacetylase Sirtuins (Hirschey et al., 2010; Fritz et al., 2012; He et al., 2012; Hebert et al., 2013; Baeza et al., 2016). IPA analysis results reveal that acetylation is related to the Sirtuin signaling pathway. Although Sirtuin family members SIRT3-SIRT5 are all located in mitochondria, SIRT3 is the main deacetylase in mitochondria (Haigis and Sinclair, 2010; Hirschey et al., 2010; Hallows et al., 2011; He et al., 2012). In SIRT3 knockout mice, the levels of mitochondrial protein acetylation in various tissues were significantly increased (Hebert et al., 2013; Dittenhafer-Reed et al., 2015). The acetylation of mitochondrial proteins can occur through two pathways, enzymes, and non-enzymatic. Enzyme acetylation involves the GNAT family of acetyltransferases, including acetyl CoA acetyltransferase (ACAT1) (Fan et al., 2014) and GCN5L1 (Scott et al., 2012; Thapa et al., 2017). Acetylation of non-enzymatic protein depends on the spontaneous transfer of acetyl group from acetyl coenzyme A to lysine residue (Baeza et al., 2015; Weinert et al., 2015), acetyl-CoA has been increasingly recognized as a substrate for protein acetylation (Glozak et al., 2005; Weinert et al., 2014). Acetylation of mitochondrial proteins mainly occurs through non-enzymatic mechanism due to there is a large amount of acetyl coenzyme A in the matrix (Baeza et al., 2015). Mitochondrial metabolism is closely related to the internal energy state of cells, and can be reversibly regulated in response to environmental pressures (Wallace, 2005). Acetylation is considered to be an ancient, conserved regulatory mechanism that links cellular metabolism to the energy state of the cell (Ahn et al., 2008). Our study indicates that lysine acetylation is a common post-translational modification in mitochondria. Previous study has confirmed that lysine acetylation is a prevalent modification in enzymes that catalyze intermediate metabolism (Zhao et al., 2010), such as the seven TCA cycle-related dehydrogenases, and oxidative phosphorylation complexes I, II, III, and V. In this study, we revealed that various enzymes involved in ATP production are highly acetylated in mitochondria, suggesting the increase in the mitochondrial function for energy production in GF mice, which exhibit antianxiety- and antidepressive-like behaviors compared with SPF mice (Zheng et al., 2016; Luo et al., 2018). Consistently, our previous studies showed the inhibition of the expression of genes related to oxidative phosphorylation pathway in gut microbiota-remodeled mice with depressive-like behaviors (Qi et al., 2020), and studies have also revealed a low cerebral bioenergetic metabolism in different brain regions, including prefrontal cortex and basal ganglia of patients with MDD (Drevets et al., 1997; Mayberg et al., 1999; Videbech, 2000; Moretti et al., 2003), which means decrease in mitochondrial function in depression. Taken together, these results indicated that mitochondrial dysfunction may be involved in the mechanisms by which gut microbiota regulates the host brain function.

The TCA cycle and oxidative phosphorylation were heavily targeted by acetylation in the present study; protein acetylation was altered in 11.0% of proteins involved in oxidative phosphorylation and 29.2% of proteins involved in the TCA cycle II pathway. All seven enzymes in the TCA cycle were acetylated, namely, dihydrolipoamide dehydrogenase (DLD), citrate synthase (CS), succinate-CoA ligase ADP-forming beta subunit (SUCLA2), succinate dehydrogenase complex flavoprotein subunit A (SDHA), oxoglutarate dehydrogenase (OGDH), aconitase 2 (ACO2), and malate dehydrogenase (MDH1). SUCLA2 is located in the mitochondrial matrix and catalyzes the conversion of succinyl-CoA and GDP to succinate and GTP (Johnson et al., 1998). SDHA is a key enzyme of the TCA cycle and respiratory complex II and has been reported to be hyperacetylated at multiple lysine residues in both mouse and human samples (Horton et al., 2016). In addition, OGDH catalyzes the conversion of 2-oxoglutarate to succinyl-CoA and CO2 within eukaryotic mitochondria, thus controlling a rate-regulating TCA cycle step. The increased expression of these enzymes would be anticipated to contribute to an acceleration of the TCA cycle (Fujisawa et al., 2016). Moreover, ACO2 plays an important role in the TCA cycle, converting citrate into its isomeric isocitrate. Our results indicated a significant increase in ACO2 acetylation in the TCA cycle. Furthermore, MDH1 is an identified protein whose acetylation pattern can be altered to accelerate the conversion of oxaloacetate into malate in the cytoplasm, thus playing a key role in TCA cycle function. The acetylation of MDH1 is one of the cross-talk mechanisms between adipogenesis and intracellular energy levels (Wang et al., 2010). Previous studies have shown that upregulated MDH1 expression can compensate for TCA energy deficiency in rats (Dong et al., 2014). Combined, these data indicate that these acetylated metabolic enzymes are upregulated in the TCA cycle. Our results therefore suggest that mice with absence of gut microbiota may have enhanced TCA cycle function.

Most cellular energy is obtained through oxidative phosphorylation, a process that requires the action of various respiratory enzyme complexes in the mitochondrial respiratory chain (Horn and Barrientos, 2008). Tissue with high energy requirements, such as the brain, contains large amounts of mitochondria and is therefore more susceptible to changes in aerobic metabolism (Boekema and Braun, 2007). Previous research has suggested that alterations in energy metabolism might be related to depression (Moretti et al., 2003). In our bioinformatic analyses, we revealed that the oxidative phosphorylation pathway was significantly acetylated in mice with absence of gut microbiota. We confirmed the upregulated expression of oxidative phosphorylation electron transport chain complex genes, such as NADH:ubiquinone oxidoreductase subunits (NDUFB4 and NDUFA5), succinate dehydrogenase (SDHA), ubiquinol-cytochrome c reductase (UQCRB, UQCR10), and ATP synthases (ATP5E, ATP5PB, ATP5PO, ATP5PD, ATP51D, ATPF1A). One ATP synthase (ATP5F) showed a reduction. Together, our results indicated that absence of gut microbiota may lead to upregulation of TCA cycle and oxidative phosphorylation in mitochondria, and revealed that the disturbances in lysine acetylation may play a regulatory role in the underlying mechanisms by which gut microbiota regulate brain function and behavioral phenotypes.

The present study has some limitations. First, because no commercially available antibodies were available against specific sites, we could not conduct functional validations of our results; thus, we will further verify the key findings in future studies. Second, in the present study, we did not conduct repeated behavioral tests. This is because the phenotype of GF mice is robust, and we have previously reported that GF mice exhibit antianxiety- and antidepression-like behaviors compared with conventionally raised SPF counterparts. Third, there was a one-to-one correspondence between proteins and modifications. We wanted to investigate changes from a modification level, so we did not detect changes in protein expression. Fourth, only male mice were used to perform the experiments and acetylated proteomic profiling in the present study. Further investigation is needed to explore whether (or how) sex-based differences might influence this model.

## Conclusion

In conclusion, we determined the lysine acetylome of the hippocampus in mice with an absence of gut microbiota. Our findings indicate that acetylated proteins are involved in a variety of biological functions and are mainly located in mitochondria. The main pathways for the regulation of lysine acetylation included the TCA cycle and oxidative phosphorylation. Importantly, several metabolic enzymes involved in the production of ATP were hyperacetylated. Overall, this study indicates that lysine acetylation alterations may play a pivotal role in mitochondrial dysfunction and may be a mechanism by which gut microbiota regulate brain function and behavioral phenotypes.

## Data Availability Statement

The datasets presented in this study can be found in online repositories. The names of the repository/repositories and accession number(s) can be found below: ProteomeXchange Consortium via the PRIDE partner repository with the dataset identifier PXD022577, https://www.ebi.ac.uk/pride/archive/projects/PXD022577/private.

## Ethics Statement

The animal study was reviewed and approved by the Ethics Committee of Chongqing Medical University.

## Author Contributions

YY, HWa, and PX designed the project. YY, XR, TC, PJ, JS, HWe, and WL implemented the animal experiments. YY, WZ, and HWa analyzed the data. YY and LL wrote the manuscript. All authors contributed to manuscript revision and consent to its publication.

## Conflict of Interest

The authors declare that the research was conducted in the absence of any commercial or financial relationships that could be construed as a potential conflict of interest.

## Footnotes

1 www.omicsbean.com:88

2 www.ingenuity.com

## Supplementary Material

Supplementary Figure 1 GO functional classification of the identified acetylated proteins.

Supplementary Table 1 Detailed information on identified acetylated peptides in mouse.

Supplementary Table 2 Motif-x analysis of the identified and quantified proteins in mouse.

Supplementary Table 3 GO classification of acetylated proteins.

Supplementary Table 4 KEGG pathway enrichment analysis.

Supplementary Table 5 Protein domain enrichment analysis.

Supplementary Table 6 Interaction network analysis.
