# Supplementary figures and images for: Proteomic Profiling of Lysine Acetylation Indicates Mitochondrial Dysfunction in the Hippocampus of Gut Microbiota-Absent Mice
Source: Front Mol Neurosci. 2021 Mar 11;14:594332. doi: 10.3389/fnmol.2021.594332 (PMC7991600; doi:10.3389/fnmol.2021.594332)

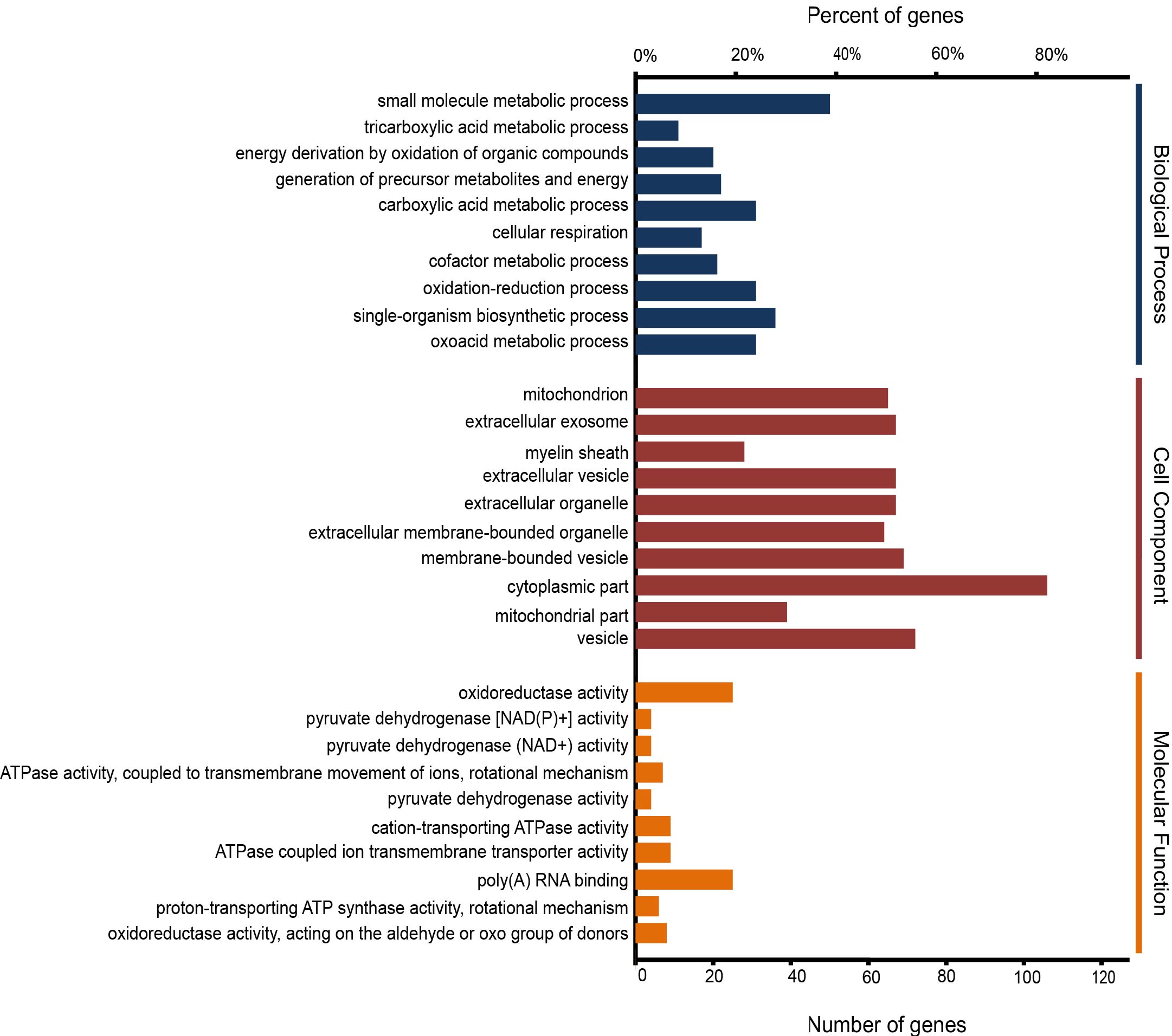

Supplement: Supplementary Figure 1 — GO functional classification of the identified acetylated proteins. [file Data_Sheet_1.ZIP › Supplementary Material/Supplementary Figure 1.jpg]
